# Revisiting the inorganic component of bone: beyond the accepted concept of “mineral”

**DOI:** 10.1038/s41598-026-64088-5

**Published:** 2026-08-05

**Authors:** Henry P. Schwarcz, Viktória K. Kis

**Affiliations:** 1 School of Earth, Environment and Society, McMaster University, Hamilton, ON Canada; 2 School of Biomedical Engineering, McMaster University, Hamilton, ON Canada; 3 HUN-REN Centre for Energy Research, Konkoly-Thege Miklós u. 29-33, Budapest, Hungary; 4 Department of Mineralogy, Eötvös Loránd University, Pázmány Péter sétány 1/C, Budapest, Hungary

**Keywords:** Apatite, X-ray diffraction, Transmission electron microscopy, Infra-red spectrometry, Definition of minerals, Materials science, Solid Earth sciences

## Abstract

Bone is widely believed to be constructed of apatite with a composition close to that of hydroxyapatite. The mineral of bone is a made up of polycrystalline 5 nm-thick plates of a monoclinic crystal with a composition like that of apatite but differing from that mineral in most significant respects. Although it is a well-defined crystalline species with diagnostic shape, size, composition range and symmetry, it is not a new mineral because it is not known to grow in any known geological environment. It appears to be a different crystalline phase from apatite*.*

## Introduction

Bone, the material from which skeletal elements are constructed in vertebrates, is principally composed of two components: collagen in the form of fibrils about 50 nm in diameter, and an inorganic crystalline phase. These crystals have traditionally been called hydroxyapatite, a calcium phosphate with the formula Ca_5_(PO_4_)_3_(OH). From Web of Science we learn that approximately 44,000 papers about bone have been published in which hydroxyapatite is referred to.

In the view of the International Mineralogical Association (IMA)^1^ all apatite-group minerals (hydroxyapatite (HA), fluorapatite, chlorapatite, etc.) are hexagonal or pseudo-hexagonal crystals which form hexagonal prisms elongated along the crystallographic **c**-axis. However, it has been shown that the inorganic component in bone is in the form of flat polycrystalline platelets, 5 ± 1 nm in thickness as determined using transmission electron microscopy (TEM), and 100 nm or more in horizontal dimensions. The crystals in these plates have monoclinic symmetry^2^. Many crystals with pseudo-hexagonal or distorted hexagonal arrangement of atoms form thin, flat flakes, for example, micas and some clay minerals. However, in most of these crystals the platy surface is oriented normal to the **c**-axis of the mineral whereas in the mineral of bone the **c**-axis has been found to be oriented in the plane of the plates, which, in turn, is in agreement with the monoclinic symmetry. The platy habit seen in bone mineral platelets (MPs) is unknown in hexagonal minerals, a fact which has been remarked upon by bone researchers^3^.

X-ray diffraction (XRD) of whole bone gives a powder pattern resembling that of apatite, a fact that led to calling bone crystals hydroxylapatite (HA). However, peaks of XRD patterns are broadened due to the small size of bone crystals, and therefore strongly overlap, obscuring the fine structure of the diffractogram. At the same time, Raman spectroscopy and NMR analysis revealed that bone crystals were poor in hydroxyl^4,5^, while spectroscopic studies showed that it contained carbonate which occupied both the hydroxyl and part of the phosphate sites^6^ as well as HPO_4_^2-^ ions substituting for PO_4_^3-^^7^. For this reason some authors have referred to the inorganic component of bone as another form of apatite^8,9^.

When collagen and other organic constituents of powdered bone are removed by treatment with bleach or another low-temperature oxidant^10^, flat platelets a few tens of nm across and about 5 nm thick are produced. This thickness is consistent with SAXS and in-tissue TEM studies^11^. Electron diffraction pattern can be obtained from these platelets by selected area electron diffraction (SAED)^12^. These patterns also resemble those of apatite.

Fast-Fourier transforms (FFT) of high-resolution transmission electron microscope (HRTEM) images of mineral from such platelets showed that the crystals in bone were monoclinic and that the flat surfaces were (010) faces of these crystals^2^. This symmetry assignment as well as the unusual morphology of bone mineral raise doubts about the identification of bone crystals as an apatite as defined by Pasero et al.^1^. Here we shall describe some further characteristics of these crystals and discuss the possibility that it is a new, yet unnamed mineral.

## Results and discussion

### Nanoscale morphology evidence

**Fig. 1:**
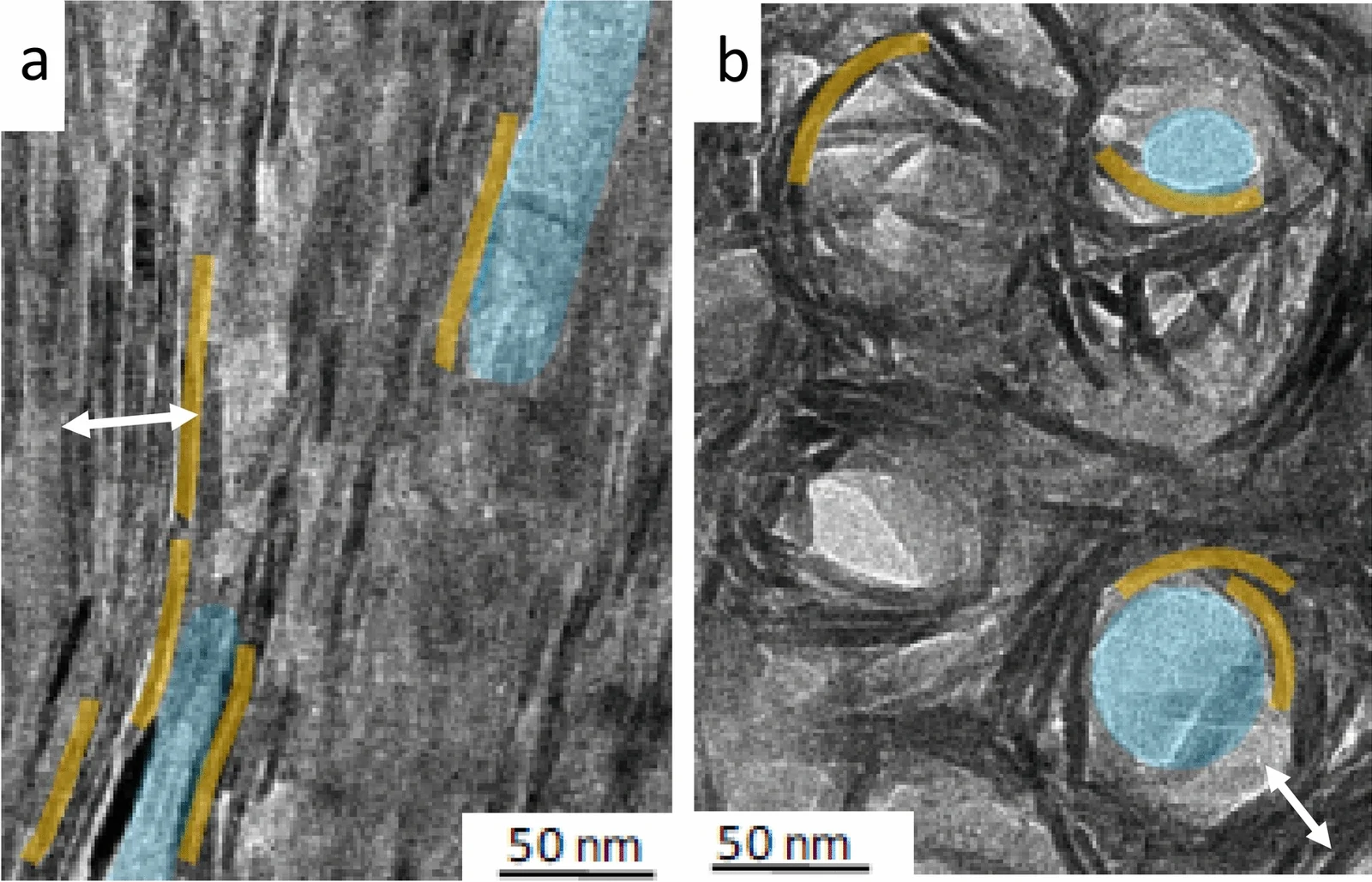
Bright field TEM images of cortical bone cut parallel (**a**) and normal (**b**) to axes of collagen fibrils. Collagen fibrils and curved mineral platelets are partially indicated with blue and yellow, respectively. White, double-headed arrows: stacks of mineral plates (adapted from Kis et al.^2^).

For convenience, we shall refer to the material which we are discussing as “bone crystals”, BC. It can be visualized in bone using TEM although it is impossible to see individual crystals. In sections of bone cut using a microtome with a diamond or steel knife, the form of plates of BC is generally distorted to the extent that it would be impossible to resolve their principal morphological characteristics^13,14^. By cutting bone with a beam of Ar^+^ or other ions in either an ion mill or a focused ion beam (FIB) device we obtain sections in which BC can be resolved. In long bones (femora, tibiae, etc.) it is possible to orient specimens in such a way that the axes of the collagen fibrils are either normal to or lie in the plane of the section. When this is done (Fig. 1 a,b) we obtain images showing the form of BC in sufficient detail to characterize it to some extent.

In Fig. 1a cut parallel to the collagen fibrils, we see that BC (the only dark to black material in the images) is in the form of elongated platelets oriented parallel to the collagen fibrils. They occur as clusters (“stacks”) of 2 to > 30 platelets spaced < 1nm apart. In Fig. 1b cut normal to the collagen fibrils we see stacks of platelets curved around the fibrils or in the spaces between them, also curved. Although each figure by itself shows only one-dimensional electron-dense objects, the combination of the two views confirms that we are seeing edgewise views of platelets about 5 nm thick. The curvature seen in Fig. 1b but not in Fig. 1a suggests that the platelets are curved into “cylindroidal” forms whose axes of curvature are oriented parallel to the fibrils^15^.

**Fig. 2:**
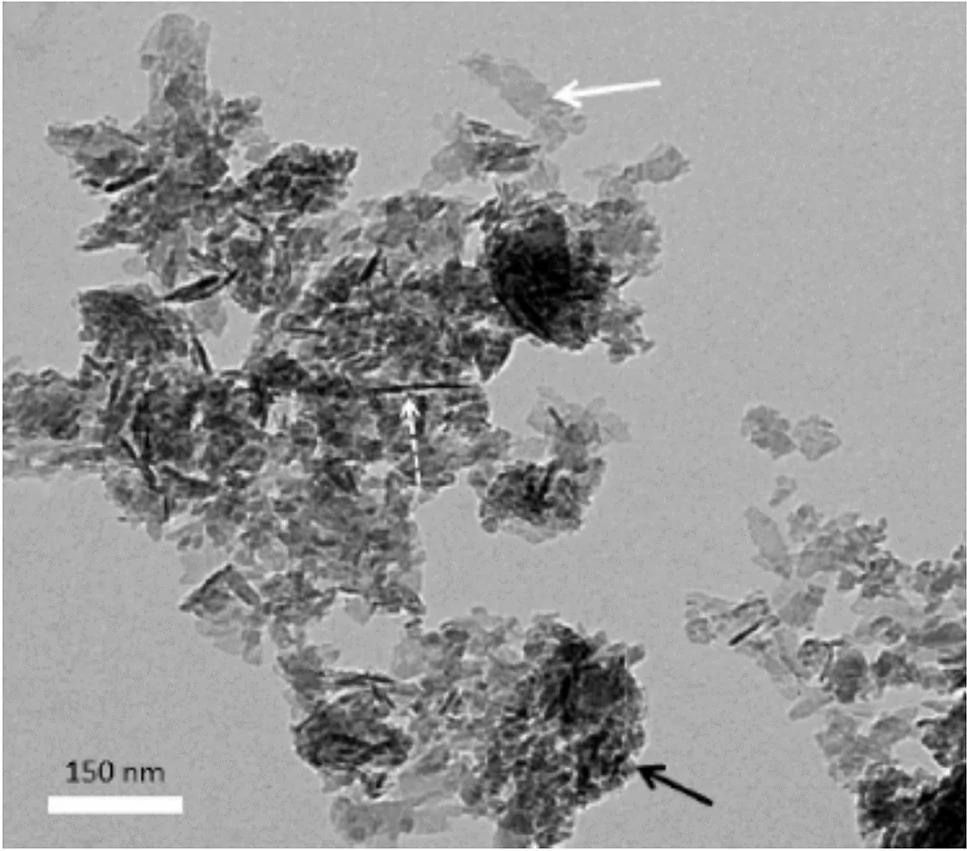
Bovine bone, powdered, and treated with NaClO to remove collagen and other organic compounds. White arrow: single mineral platelet, lying flat. Dashed arrow: single mineral platelet, lying edgewise on grid. Black arrow: clump of many platelets adhering to each other; scale = 150 nm. From Pang et al.^16^.

Figure 2 shows typical examples of mineral platelets isolated by treatment of powdered bone with bleach. When removed from bone, the platelets lie flat or stand on edge on the carbon film used for TEM viewing, showing that their curvature inside bone is entirely due to their confinement in curved spaces around or between collagen fibrils^15^.

**Fig. 3:**
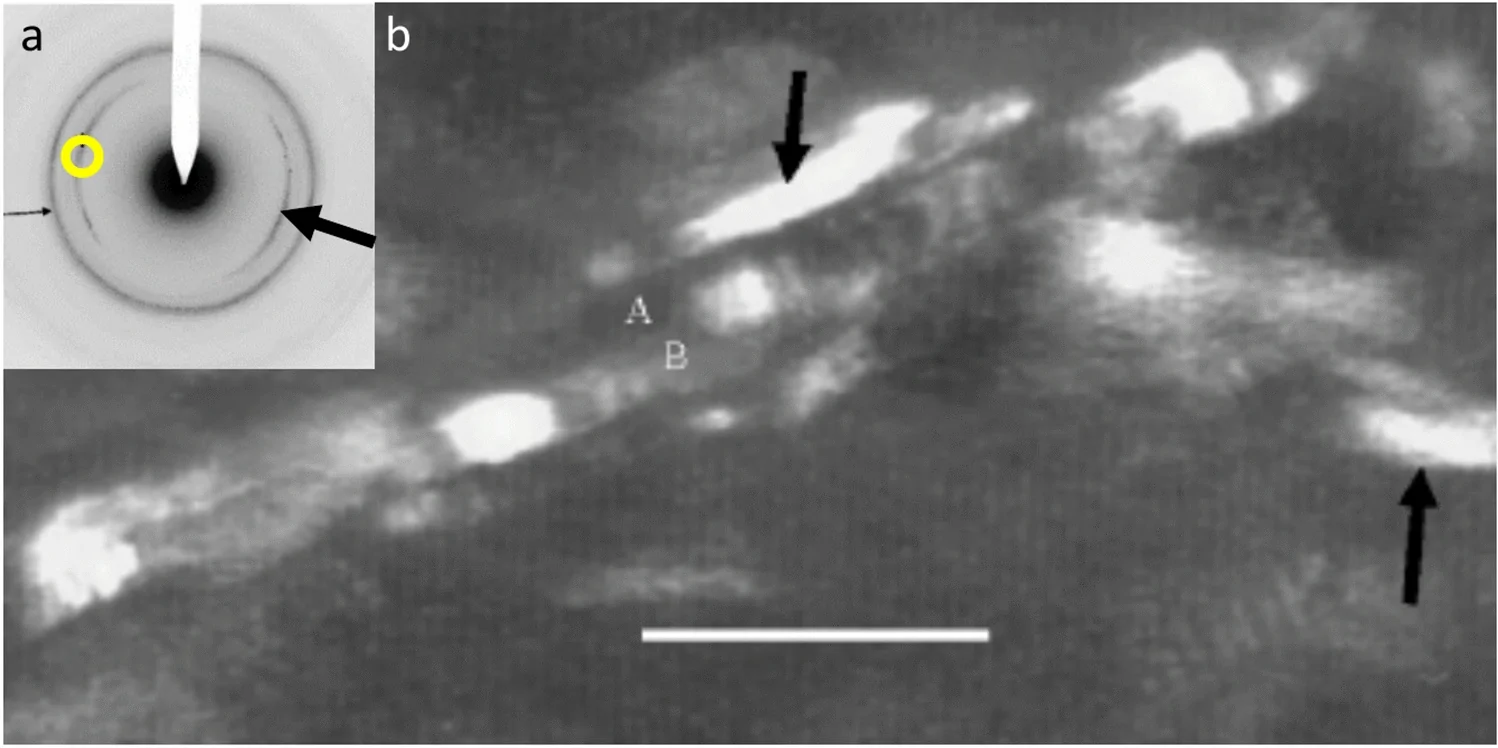
(**a**) SAED pattern from TEM section of human cortical bone oriented parallel to collagen fibrils; arrow = 002 arc; (**b**) Dark field images of mineral platelets in a section cut parallel to collagen fibrils. Stack of three mineral plates viewed in dark-field using electrons from yellow circle on 002 arc; white (illuminated) volumes are single crystals of BM. Beside them are other crystals in different orientation which do not scatter in the orientation selected by the particular position of the aperture on the 002 arc. Scale = 25 nm.

Using electrons scattered by the (002) planes in a SAED pattern (Fig. 3a), we can construct dark field (DF) images of TEM sections of bone. In these images only those parts of the mineral platelet will appear bright, which scatter electrons from the part of the 002 diffraction arc which was selected for imaging (see yellow circle on Fig. 3a). Dark field images show that within the mineral platelets there are single crystals about 30 nm in length and of the same thickness as the platelet (~ 5 nm). That is, each platelet is composed of several nanocrystals, attached laterally.

The average length measured on DF images is about 30 nm consistent with Scherrer measurements of XRD patterns from powdered bone^11^. The thickness of the MPs in TEM sections averages around 5 nm but varies significantly (3 – 6 nm;^14^).

Careful examination of FFT patterns from high-resolution TEM (HRTEM) images of plates obtained by bleach-treatment of powdered bone also shows that they are composed of multiple crystals tilted at small, varying angles with respect to each other^2^. FFT patterns also show that the **c**-axis is lying in the plane of the plate in agreement with Moradian-Oldak et al.^12^, and that on average, it is pointing towards the longest dimension of the mineral plate. We refer to the plates as mesocrystals, in which multiple crystals are arranged together, usually with small misalignment between the crystal components^17^.

**Fig. 4:**
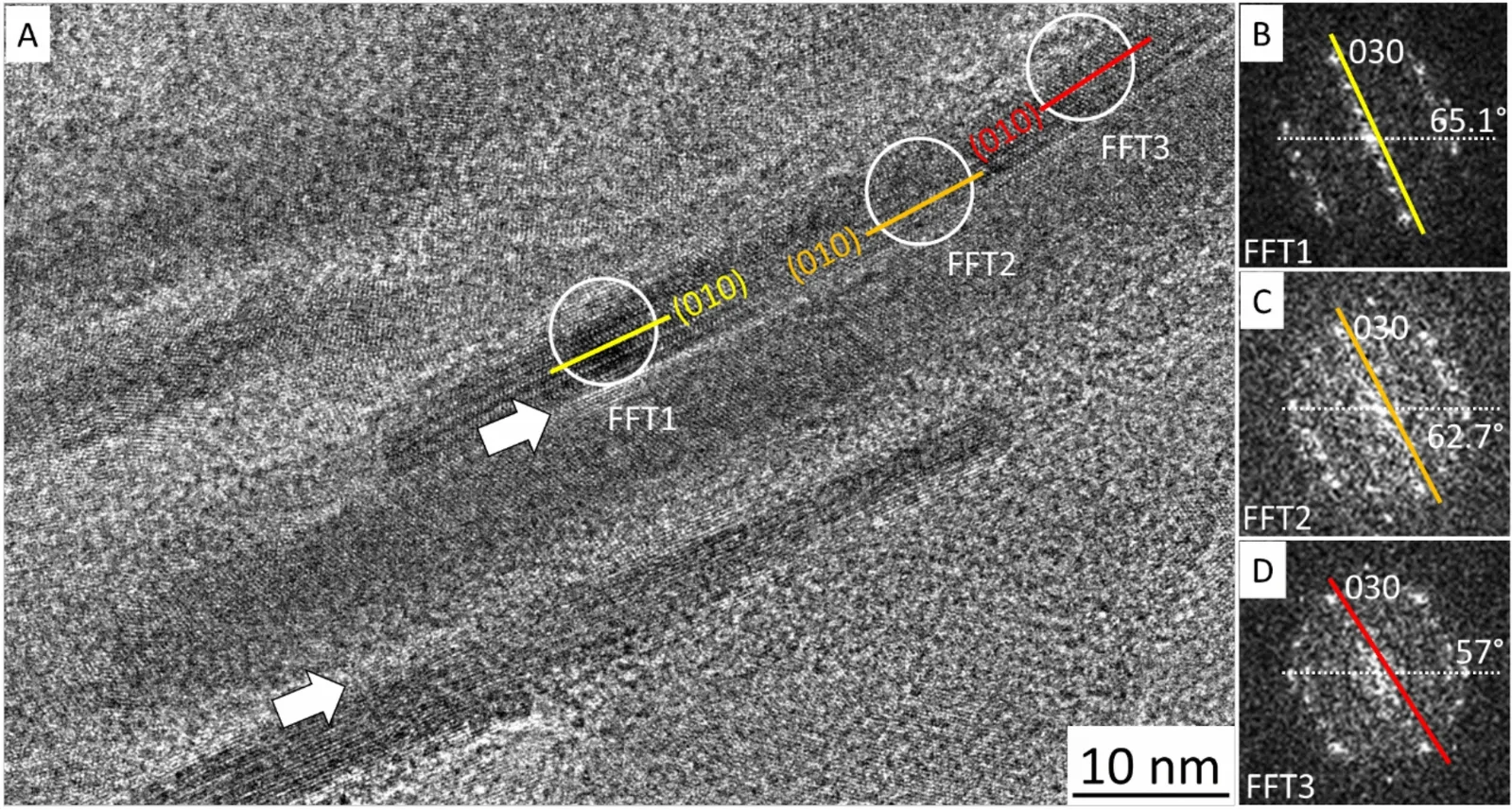
(**A**) HRTEM image of adjacent mineral platelets in bone tissue. (**B**-**D**) Nanocrystal orientation is analyzed inside a single platelet. Separate FFTs taken from areas of 6.5 nm diameter show a gradual rotation of the dominant (010) planes. The approximate orientation of this mineral platelet is [201]_hex-mono_. White arrows on A show gaps between platelets in a stack.

TEM images of ion-beam milled sections of bone cut parallel to the fibril axes allow us to see that neighboring mineral platelets, and also successive sub-volumes inside mineral plates exhibit slightly different orientations of the crystals. HRTEM views of such a section (Fig. 4) show that their **c**-axes are approximately co-aligned.

The size of the mesocrystalline platelets liberated during treatment with bleach varies widely but has not yet been carefully measured. They are elongated and range from a few nm to > 100 nm in maximum dimension. In edgewise views of mineral platelets in TEM sections of bone cut parallel to the collagen fibrils they range up to > 200 nm in length^14^. It is likely that during the powdering of bone prior to bleaching these plates have been partly broken, so that plates in images like Fig. 2 are mostly fragments of larger plates.

The only well-developed crystal faces are the flat surfaces of the plates formed as a result of the fusion of several component nanocrystals. According to FFT analysis, this flat surface is the (010) face of the crystal, in agreement with SAED observation^12^. The external edges of the plates as seen mounted on carbon films are sometimes straight for short distances suggesting the presence of a crystal face but too limited in scale to permit evaluating any angles. Although too small to be resolved optically, polycrystalline plateles of BC can be poorly resolved in SEM images^18,19^.

In sections cut perpendicular to the collagen fibrils, mineral platelets are mostly in [001] zone axis orientation, that is, the [001] zone axes of the nanocrystals are in line or very close to the electron beam. This relative orientation of the crystal and the electron beam produce strong diffraction contrast, and as a result the mineral platelets appear dark. When they are tilted away from this orientation, diffraction contrast decreases, and thus mass-thickness contrast dominates, causing mineral platelets to be only dimly perceptible as shown on HRTEM images of regions between stacks of plates^15^.

### Chemical composition of bone crystals

The gross composition of BC as determined by chemical analysis of deproteinized whole bone is approximately uniform regardless of the origin of the skeletal element or the animal^20,21^. The Ca/P ratio is slightly lower than 1.67, the expected ratio in stoichiometric HA. Small concentrations (< 1 mol%) of Na^+^ and Mg^2+^ are present as well as trace amounts of Sr^2+^, Pb^2+^, and other heavy metals present as trace constituents of diet. Detailed analysis using µm-scale Fourier transform infrared (FTIR) analysis shows that HPO_4_^2-^ substitutes for PO_4_^3-^ to varying extents in different regions of the same section of bone although the resolution is not sufficient to say if adjacent platelets or crystals within platelets might vary^22^. Micro-FTIR also reveals the presence of CO_3_^2-^ ions substituting for both for PO_4_^3-^ and at the OH^-^ site^23^. NMR measurements showed that BC contains at most 20% of the OH^-^ ions expected in stoichiometric HA^4^, confirming the absence of OH^-^ inferred from Raman spectroscopy^5^. The main difference between the compositions of HA and BC is their complex anion content, therefore, BC should not be called hydroxylapatite^24^. A general formula for BC if it has the same stoichiometry as HA, is.

$${\mathrm{C}\mathrm{a}}_{10-\mathrm{x}},{\square}_{\mathrm{x}}[{\left({\mathrm{P}\mathrm{O}}_{4}\right)}_{6-\mathrm{x}}{( {\mathrm{H}\mathrm{P}\mathrm{O}}_{4}^{2-}, {\mathrm{C}\mathrm{O}}_{3}^{2-})}_{\mathrm{x}}]{({\mathrm{O}\mathrm{H}}_{1-\mathrm{y}} {\{{\mathrm{C}\mathrm{O}}_{3}\}}_{\mathrm{y}})}_{2-\mathrm{x}},{\square}_{\mathrm{x}}$$.

For example, a composition of Ca_7.5_(PO_4_)_2.8_(HPO_4_)_2.6_(CO_3_)_0.6_(OH)_0.2_ was provided for a sample of sheep bone studied by^2^^,^^25^.

### Comparison with apatite

Observations of their monoclinic crystal symmetry^2^ coupled with the plate-like form of bone mineral are strong indications that they are not crystals of the mineral apatite, in the sense used by the IMA^1^. Almost all apatite is found to be a hexagonal mineral with crystal symmetry P6_3_/m. Pure hydroxylapatite similarly to chlorapatite end-member, can also crystallize in the monoclinic space group P2_1_/b^26,27^. In this case, the monoclinic symmetry is produced by alternating upward and downward pointing hydroxyl anions in neighbouring structural channels, which run parallel to **c**-axis. This arrangement requires stoichiometric amount of hydroxyl in the structure. Most geological apatites which are apparently single crystals, are solid solutions of end member (OH^-^, Cl^-^, F^-^)-apatites. Because distribution and arrangement of channel anions is random, they seem hexagonal when studied with X-ray diffraction. Accordingly, single crystals of naturally occurring mesoscopic hydroxyapatite from Holly Springs^28^ were determined to be in the P6_3_/m space group^29^, which is in agreement with its fluoride and chloride content^30^.

According to equilibrium morphology calculations, pure monoclinic hydroxyapatite tends to adopt pseudo-hexagonal habit via twinning^31^, which makes it hard to distinguish from the hexagonal polymorph^27^. Such morphology fits into the definition of apatite required by the IMA^1^. Akizuki et al.^32^ report optical zonation of geological apatite of (pseudo)hexagonal morphology and explain it with F-/OH- ordering. Monoclinic hydroxyapatite single crystals have been grown synthetically by replacement of Cl^-^ ions in a chlorapatite at 1200 °C^33^ and in the presence of citrate at 135 °C^34^.

Crystal structure determination of bone apatite nanocrystals in polycrystalline mineral platelets is a challenge because of their small dimensions and mesocrystalline nature. The monoclinic unit cell proposed by us^2^ explains the flat crystal shape and main structure features.

No other naturally occurring apatite crystals have the crystal habit observed in bone, namely polycrystalline plates ~ 5nm thick and a few tens to hundreds of nm across, with the **c**-axes lying in the flat plane. Based on the monoclinic crystal symmetry and habit we conclude that the mineral in bone is not an apatite in the sense provided by IMA^1^.

According to Nickel and Grice^35^, “if the crystal structures of the polymorphs have essentially the same topology, differing only in terms of a structural distortion” they should not be regarded as separate mineral species but “can be distinguished by the addition of crystallographic suffixes to the mineral name”. In case of bone crystals, based on their diagnostic compositional range, namely the high CO_3_^2-^ and HPO_4_^2-^ and limited OH^-^ content we conclude that they are not polymorph of monoclinic hydroxylapatite. Shape and HRTEM features are consistent with monoclinic structure. However, the lack of stoichiometric OH^-^ calls into question the applicability of hydroxylapatite as a mineral model for BC^24^ and implies that BC is monoclinic for different reason than monoclinic hydroxylapatite^33^.

### Occurrence of BC

BC is the principal crystalline component in all bone and dentin. It is not present in any other sites in vertebrates and is also are not present in any invertebrates. It is not yet known to occur in any other non-biological setting. In vertebrates, other Ca phosphates have been found to occur including HA (in dental enamel), amorphous Ca phosphate in incipient enamel^36^, and octacalcium phosphate (OCP) in dental calculus^37^. None of these phases occur in bone. Octacalcium phosphate was suggested to be the main mineral in bone^38^ but it is not detected in X-ray diffraction or HRTEM analyses of bone.

Crystals of calcium phosphate resembling BC have been synthesized in various experimental syntheses of “apatite” by Bertinetti et al.^39^, Iafisco et al.^40^, Nassif et al.^41^, Schwarz and Epple^42^, and Vandecandelaere et al.^43^. These have been grown in aqueous solutions in contact with atmospheric levels of CO_2_ at temperatures less than 40⁰C. Crystals grown at higher temperatures (> 50⁰C) from solutions of similar chemistry (Ca^2+^, PO_4_^3-^, and pH ~ 7) are hexagonal, six-sided crystals of apatite (e.g., Ferraz et al.^44^).

### Is BC a mineral?

We have shown that the crystalline Ca phosphate phase of bone does not fit the apatite concept of the IMA. The question arises: is it a mineral? According to Nickel and Grice^35^ “A mineral substance is a naturally occurring solid that has been formed by geological processes”. The crystals of Ca phosphate in bone cannot be a new mineral because they have never been found formed by a geological process. However, BC forms naturally in all vertebrates and has well defined range of composition and structure. Moreover, its crystals have a well-defined habit with respect to size and crystal faces and overall shape, which does not form under other known non-laboratory conditions.

Other crystalline materials exist which are almost unknown except for their occurrence in animals. Calcium oxalate occurs as two minerals, weddellite (CaC_2_O_4_·(2 + x)H_2_O) and whewellite (CaC_2_O_4_·H_2_O) which occur as kidney stones, but both have also been found in geological occurrences. Crystal structure, composition and crystal habit are the same for the geological and animal crystals. Some crystals found in humans (e.g., calcium stearate, calcium palmitate) occur as concretions in the bile duct but are not minerals.

Nickel and Grice^35^ write that “purely biogenic substances that have no geological counterparts, or whose origin owes essentially nothing to geological processes, are not regarded as minerals. However, substances formed by the action of geological processes on organic material, such as the chemical compounds crystallized from organic matter in shale or from bat guano, can be accepted as minerals”. Since no such geological occurrence has yet been observed of crystals resembling BC, we must conclude that it is not a mineral.

## Conclusions: A polymorph of calcium phosphate

The crystalline phase that constitutes bone is a calcium phosphate with a chemical formula like that of apatite. It cannot be referred as a monoclinic polymorph of hexagonal hydroxylapatite, because of substantial difference in composition. An analogous situation exists for calcium carbonate which occurs in rocks and organisms in at least two forms: aragonite which is orthorhombic, and calcite which is hexagonal. Calcite and aragonite are two polymorphs of calcium carbonate, both with the same chemical formula. Similarly, silicon dioxide (SiO_2_) occurs in rocks as at least four different polymorphs: quartz, tridymite, cristobalite, and coesite. Many minerals occur as two or more polymorphic forms and each is given a different name.

No geological setting of BC is known. Therefore (according to^35^), we may not refer to it as a mineral. We must therefore discontinue the use of the word “mineral” to describe BC and must refer to it as “the crystalline material in bone”.

Our bone mineral structure model^2^ is consistent with nanocrystal morphology and with the orientation relationship between bone apatite and collagen^3^ and is further supported by crystal shape calculations^27,31^. This provides an alternative and more comprehensive nanoscale understanding of bone crystals.

## Methods

HRTEM investigation was performed using a Thermo Fischer Themis G2 Cs corrected TEM (Schottky FEG, point resolution in HRTEM mode 0.7 nm). Bone samples were prepared using Ar^+^ ion beam milling, with LN_2_ cooling. An amorphous carbon layer was evaporated onto the sample surface to avoid charging.

Samples of bone presented are as follows: Figs. 1 and 4: cryo-Ar milled sections of cortical bone from human femur, provided by Prof. Z. Szekanecz, University of Debrecen, Hungary. Figure 2: crushed fragments of the bone of mature castrated male cow (steer) obtained from a butcher shop in Hamilton, ON, Canada. Figure 3: a cryo-Ar ion-milled section of the femoral diaphysis of a healthy 60 years old human male remaining from an allograft procedure. The bone was obtained from a service that provided samples of human bone for scientific and medical investigations.

## Data Availability

Data are available upon request.
